# Transcriptome analysis of bitter acid biosynthesis and precursor pathways in hop (*Humulus lupulus*)

**DOI:** 10.1186/1471-2229-13-12

**Published:** 2013-01-24

**Authors:** Shawn M Clark, Vinidhra Vaitheeswaran, Stephen J Ambrose, Randy W Purves, Jonathan E Page

**Affiliations:** 1 National Research Council of Canada, 110 Gymnasium Place, Saskatoon, SK, S7N 0W9, Canada; 2 Department of Biology, University of Saskatchewan, 112 Science Place, Saskatoon, SK, S7N 5E2, Canada

## Abstract

**Background:**

Bitter acids (*e.g.* humulone) are prenylated polyketides synthesized in lupulin glands of the hop plant (*Humulus lupulus*) which are important contributors to the bitter flavour and stability of beer. Bitter acids are formed from acyl-CoA precursors derived from branched-chain amino acid (BCAA) degradation and C5 prenyl diphosphates from the methyl-D-erythritol 4-phosphate (MEP) pathway. We used RNA sequencing (RNA-seq) to obtain the transcriptomes of isolated lupulin glands, cones with glands removed and leaves from high α-acid hop cultivars, and analyzed these datasets for genes involved in bitter acid biosynthesis including the supply of major precursors. We also measured the levels of BCAAs, acyl-CoA intermediates, and bitter acids in glands, cones and leaves.

**Results:**

Transcripts encoding all the enzymes of BCAA metabolism were significantly more abundant in lupulin glands, indicating that BCAA biosynthesis and subsequent degradation occurs in these specialized cells. Branched-chain acyl-CoAs and bitter acids were present at higher levels in glands compared with leaves and cones. RNA-seq analysis showed the gland-specific expression of the MEP pathway, enzymes of sucrose degradation and several transcription factors that may regulate bitter acid biosynthesis in glands. Two branched-chain aminotransferase (BCAT) enzymes, *HlBCAT1* and *HlBCAT2*, were abundant, with gene expression quantification by RNA-seq and qRT-PCR indicating that *HlBCAT1* was specific to glands while *HlBCAT2* was present in glands, cones and leaves. Recombinant HlBCAT1 and HlBCAT2 catalyzed forward (biosynthetic) and reverse (catabolic) reactions with similar kinetic parameters. HlBCAT1 is targeted to mitochondria where it likely plays a role in BCAA catabolism. HlBCAT2 is a plastidial enzyme likely involved in BCAA biosynthesis. Phylogenetic analysis of the hop BCATs and those from other plants showed that they group into distinct biosynthetic (plastidial) and catabolic (mitochondrial) clades.

**Conclusions:**

Our analysis of the hop transcriptome significantly expands the genomic resources available for this agriculturally-important crop. This study provides evidence for the lupulin gland-specific biosynthesis of BCAAs and prenyl diphosphates to provide precursors for the production of bitter acids. The biosynthetic pathway leading to BCAAs in lupulin glands involves the plastidial enzyme, HlBCAT2. The mitochondrial enzyme HlBCAT1 degrades BCAAs as the first step in the catabolic pathway leading to branched chain-acyl-CoAs.

## Background

**Figure 1:**
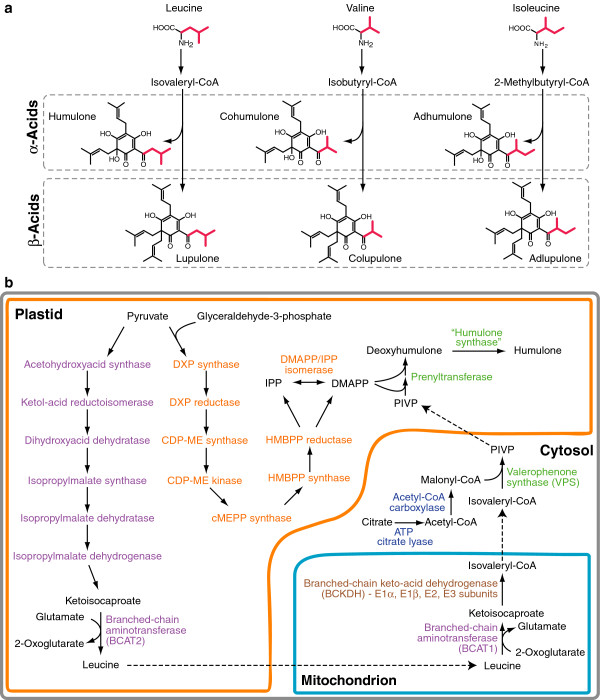
**Biosynthesis of hop bitter acids from branched-chain amino acid (BCAA) precursors.a**) The acyl side-chains of humulone/lupulone are derived from leucine, cohumulone/colupulone from valine and adhumulone/adlupulone from isoleucine via the corresponding acyl-CoA thioesters. The carbon chain of the BCAAs is shown in red to indicate their incorporation into the α- and β-acids. **b**) Schematic depiction of the major pathways contributing to the biosynthesis of the major α-acid product, humulone, in lupulin glands. Pathways are coloured to match Figure 2b: BCAA biosynthesis, purple; BCKDH complex, brown; MEP pathway, orange; malonyl-CoA biosynthesis, blue; bitter acid biosynthesis, green. The movement of metabolic intermediates between cellular compartments is indicated by dashed line. Note: the subcellular localization of the putative humulone synthase enzyme is not known.

The female inflorescences (‘cones’) of *Humulus lupulus* L. (hop, Cannabaceae) contain prenylated acylphloroglucinols and prenylchalcones (e.g. xanthohumol) that are important for the brewing industry and have potential medicinal uses [1-3]. The acylphloroglucinols, referred to as bitter acids because they contribute the characteristic bitter flavour of beer, include the α-acid humulone and its acyl-side chain variants cohumulone and adhumulone (Figure 1a). Antimicrobial β-acids (e.g. lupulone, colupulone and adlupulone), which differ from α-acids by the presence of an additional dimethylallyl prenyl group, are also abundant. Hop cultivars vary widely in their content and composition of bitter acids with some “super-alpha” cultivars containing greater than 20% humulone by dry weight in cones [4].

The bitter acids and other prenylated polyketides occur mainly in lupulin glands, which are glandular trichomes present on hop cones and leaves [5]. Glandular trichomes typically have little or no photosynthetic activity and often contain only a few highly active biosynthetic pathways, which has made transcriptome analysis of isolated trichome secretory cells useful for the identification of the genes involved in the production of specialized metabolites [6,7]. Enzymes involved in the biosynthesis of xanthohumol and terpenoids in hop lupulin glands [5,8] and cannabinoids in glandular trichomes of the closely related species *Cannabis sativa* (Cannabaceae) [9,10] have been identified using a combination of trichome-targeted EST analysis and biochemical assays.

The precursors for bitter acid biosynthesis are branched-chain acyl-CoAs thioesters, which function as primers for polyketide synthesis, malonyl-CoA, which is the polyketide extender and dimethylallyl diphosphate (DMAPP), which serves as a prenyl donor. The acyl-CoAs, isovaleryl-CoA, isobutyryl-CoA and 2-methylbutyryl-CoA, are obtained by the degradation of the branched-chain amino acids (BCAA) leucine, valine and isoleucine, respectively (Figure 1a). The study of BCAA metabolism is of particular interest in hop since it is one of the few plants that produces large amounts of BCAA-derived natural products in specialized secretory structures. Some Solanaceae species utilize BCAAs in the trichome-localized production of acyl sugars [11,12].

BCAA biosynthesis in plants consists of eight enzymatic steps to produce leucine and valine from pyruvate, or isoleucine from threonine and pyruvate (Figure 1b) (reviewed in Binder *et al.*[13]). Branched-chain aminotransferase (BCAT) enzymes catalyze both the final step in BCAA biosynthesis and the first step in BCAA degradation. These two phases are physically separated, with biosynthesis occurring in plastids and catabolism in mitochondria. BCATs have been studied in both Arabidopsis and tomato with each containing six and seven isoforms, respectively [14-16]. BCAA derived CoA-esters are generated by the branched-chain ketoacid dehydrogenase complex (BCKDH) which is made up of four subunits [17]. Three of these subunits, ketoacid dehydrogenase E1α and E1β, and dihydrolipoyl acyltransferase (E2), are specific to the BCKDH complex while the dihydrolipoyl dehydrogenase (E3) subunit also functions in other metabolic pathways [18].

Labeling studies have shown that the DMAPP required for the prenylation of bitter acids is provided by the methyl-D-erythritol 4-phosphate (MEP) pathway localized in plastids [19]. MEP-derived isoprenoids are also used in the biosynthesis of mono- and sesquiterpenes, which co-occur with bitter acids in lupulin glands [8]. The MEP pathway is initiated with glyceraldehyde-3-phosphate and pyruvate; the latter is also used in BCAA biosynthesis (Figure 1b). Previous investigations have shown that transcripts for the seven MEP pathway enzymes and DMAPP/IPP isomerase are abundant in lupulin glands but their expression was not compared with leaves or other organs [5,8].

The final steps in bitter acid biosynthesis are unique to hop. The type III polyketide synthase enzyme valerophenone synthase (VPS) condenses a BCAA-derived acyl-CoA starter molecule with three molecules of malonyl-CoA to form the polyketide core (e.g. phlorisovalerophenone, PIVP) [20] (Figure 1b). One or more aromatic prenyltransferase enzymes perform two C5 prenylations to yield diprenyl intermediates (e.g. deoxyhumulone) [21]. At this point the α- and β-acid pathways diverge with a third prenylation forming the β-acids while an oxygenation reaction, catalyzed by an as yet unknown humulone synthase enzyme, yields the α-acids [22].

Here we used RNA-seq to assemble and compare the transcriptomes of lupulin glands, cones (with glands removed) and leaves. We analyzed the expression of genes involved in precursor biosynthesis, including BCAAs, MEP-derived isoprenoids and malonyl-CoA, and the final steps in bitter acid formation. We focused on the BCAT enzymes which play key roles in the formation of branched-chain acyl-CoAs. Two BCAT-encoding genes were present, *HlBCAT1* and *HlBCAT2*, with transcripts for both more abundant in lupulin glands compared with leaves and cones. HlBCAT1 and HlBCAT2 were experimentally-localized using GFP fusion proteins to the mitochondrion and plastid, respectively. Assays of recombinant HlBCAT1 and HlBCAT2 showed they catalyze biosynthetic and catabolic reactions. These findings provide evidence for lupulin gland-specific BCAA and isoprenoid metabolism to produce precursors for bitter acid biosynthesis.

## Results

### Transcriptome sequencing and assembly

Lupulin glands were isolated from the mature hop cones of four commercial cultivars grown for their high α-acid content: ‘Taurus’, ‘Nugget’, ‘Magnum’ and ‘Apollo’. Leaves and cones were also collected from ‘Taurus’ and ‘Apollo’ cultivars. The cone samples consisted of the photosynthetic bracts and bracteoles that remained after the removal of lupulin glands. RNA purified from eleven samples (five gland, three cone, three leaf) was reverse-transcribed and the cDNAs sequenced on a single lane of an Illumina Hiseq 2000 instrument as a multiplexed sample. Each cDNA produced 15.7-24.7 million reads (Additional file 1: Table S1). The reads obtained from all samples were assembled using Trinity [23] and refined using Cdhit-EST [24] to produce a transcriptome of ~170 000 contigs with an average read length of 745 bp. This transcriptome was used as the reference for RNA-seq analysis of the individual tissues using CLC bio. At least 87% of the reads from each sample were mapped to the reference transcriptome for each tissue (Additional file 1: Table S1).

### Comparison of metabolic transcripts in lupulin glands, cones and leaves

**Figure 2:**
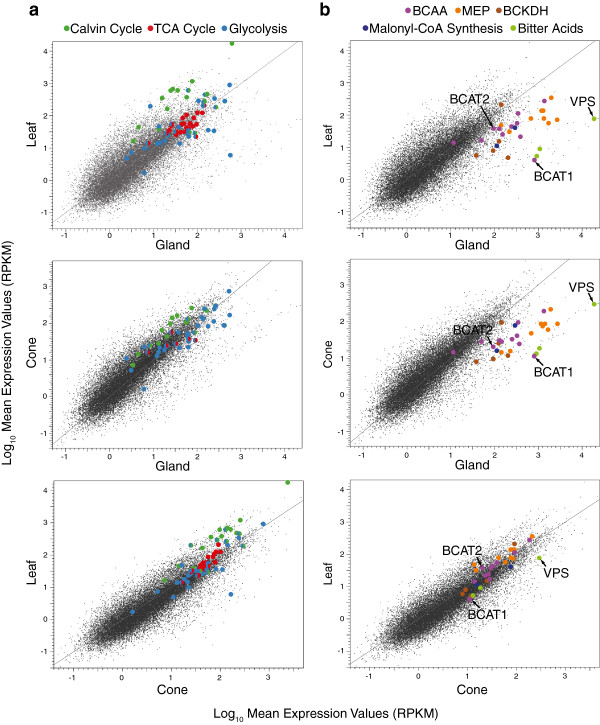
**Scatter plot comparison of gene expression of metabolic pathways between lupulin glands, leaves and cones.** Data is presented as reads per kb exon model per million mapped reads (RPKM). Corresponding gene names and expression values can be found in Table S2. **a**) Expression of genes encoding enzymes of primary metabolic pathways (Calvin cycle, TCA cycle and glycolysis). **b**) Expression of genes encoding enzymes of BCAA metabolism (BCAA, BCKDH complex, *HlBCAT1* and *HlBCAT2*), MEP pathway, malonyl-CoA synthesis and bitter acid biosynthesis.

We compared the expression of mRNAs encoding primary metabolic enzymes (Calvin cycle, tricarboxylic acid (TCA) cycle and glycolysis) between the three tissues (Figure 2a, Additional file 2: Table S2). As expected, Calvin cycle genes were predominantly represented in the photosynthetic tissue (leaves and cones) but not in the lupulin glands. Notable exceptions were the Calvin cycle enzymes involved in glycolysis, which are important for the flow of carbon from sucrose to pyruvate in the glands. An isoform of the Rubisco small subunit was one of the most abundant genes in the leaf transcriptome, while the Rubisco large subunit was not highly expressed. TCA cycle genes showed only minor differences in expression levels between the three tissues. Transcripts encoding glycolytic enzymes were also represented at comparable levels in leaves and lupulin glands, however, some genes within this pathway showed specificity to one tissue over the other. Several genes encoding enzymes required for sucrose utilization (e.g. *fructokinase*, *neutral alkaline/invertase* and *sucrose synthase*) were more highly expressed in the gland versus leaf tissue.

MEP pathway genes were more highly expressed in lupulin glands compared with green tissues. As expected from the need for malonyl-CoA for polyketide synthesis, transcripts encoding acetyl-CoA carboxylase [25] and ATP-citrate lyase [26] were abundant in the glands (Figure 2b).

Most genes in the BCAA biosynthetic pathway showed higher expression in lupulin glands compared to cones and leaves (Figure 2b), with the transcript for ketol-acid reductoisomerase showing very high abundance. Exceptions were the low expression of threonine deaminase, which is only required for isoleucine biosynthesis, and the regulatory subunit of acetohydroxyacid synthase, which had minimal expression in all three tissues (Additional file 2: Table S2). Four putative BCAT-encoding genes were identified in the transcriptome. Two of these genes, denoted *Humulus lupulus BCAT1* (*HlBCAT1*) and *HlBCAT2*, were abundant in the lupulin glands and *HlBCAT1* showed gland-specific expression (Figure 2b). Formation of the BCAA derived CoA-esters is carried out by the BCKDH complex (Figure 1b). Two isoforms of the E1α subunit were identified in the dataset and the expression of both isoforms along with the E1β and E2 subunits were increased in the lupulin glands (Figure 2b). The expression of the E3 subunit was similar in all tissues.

*VPS* was one of the most abundant mRNAs in the lupulin gland dataset, dwarfing all other genes involved in secondary metabolism examined here (Figure 2b). Two aromatic prenyltransferase genes, the previously characterized *HlPT1*[21] and *HlPT2*, showed similar gland-specific expression.

### Lupulin gland-specific transcription factors

We also analyzed the RNA-seq data for transcription factors that may be involved in regulating bitter acid biosynthesis or other lupulin gland-specific metabolic activities. Forty-eight transcription factors were significantly more abundant in lupulin glands compared to the leaves (Additional file 3: Table S3). Of these, a subset of 29 was also higher in glands compared to cones. Eleven transcription factors were expressed at more than ten-fold higher levels in the lupulin glands compared to cones and leaves (Table 1).

**Table 1:** Transcription factors that are ten-fold more abundant in the lupulin glands compared to leaves and cones

**Feature ID**	**Description**	**Fold change**	
		**Gland**^a^**vs Leaf**^b^	**Gland**^a^**vs Cone**^b^
comp76122_c0_seq4	MYB	498.2	21.63
comp50231_c0_seq1	WRKY	138.5	30.8
comp68974_c0_seq1	WRKY	127.2	34.3
comp53904_c0_seq1	MYB (CAN883 homolog^c^)	75.6	14.5
comp69082_c0_seq1	MYB	67.3	41.5
comp70463_c0_seq1	AP2-like ethylene response	56.6	36.4
comp58890_c0_seq1	Homeobox-leucine zipper	54.9	23.0
comp27968_c0_seq1	Zinc finger	50.0	10.1
comp72691_c0_seq1	Homeobox-leucine zipper	45.3	16.7
comp73886_c0_seq1	WRKY	39.9	23.4
comp63432_c0_seq1	MYB (CAN738 homolog^c^)	18.8	23.1

Fold change represents the difference between mean RPKM values for gland compared to leaf and cone.

^a^ n=5.

^b^ n=3.

^c^ Marks *et al.*[38].

### Validation of gene expression by quantitative RT-PCR

The RNA-seq analysis of the BCKDH complex, lupulin gland-specific BCATs, VPS and HlPT1 was confirmed using qRT-PCR. *HlBCAT1* was lupulin gland specific while *HlBCAT2* was expressed in both lupulin glands and leaves (Additional file 4: Figure S1). *E1α*, *E1β* and *E2* of the BCKDH complex were more highly expressed in lupulin glands while the E3 subunit was represented equally in all tissues. The genes encoding *VPS* and *HlPT1* were highly expressed in the lupulin glands while essentially non-existent in the leaf tissue. All of these findings were in agreement with the RNA-seq data (Figure 2b).

### Metabolite profiling of BCAAs, acyl-CoAs and bitter acids

**Figure 3:**
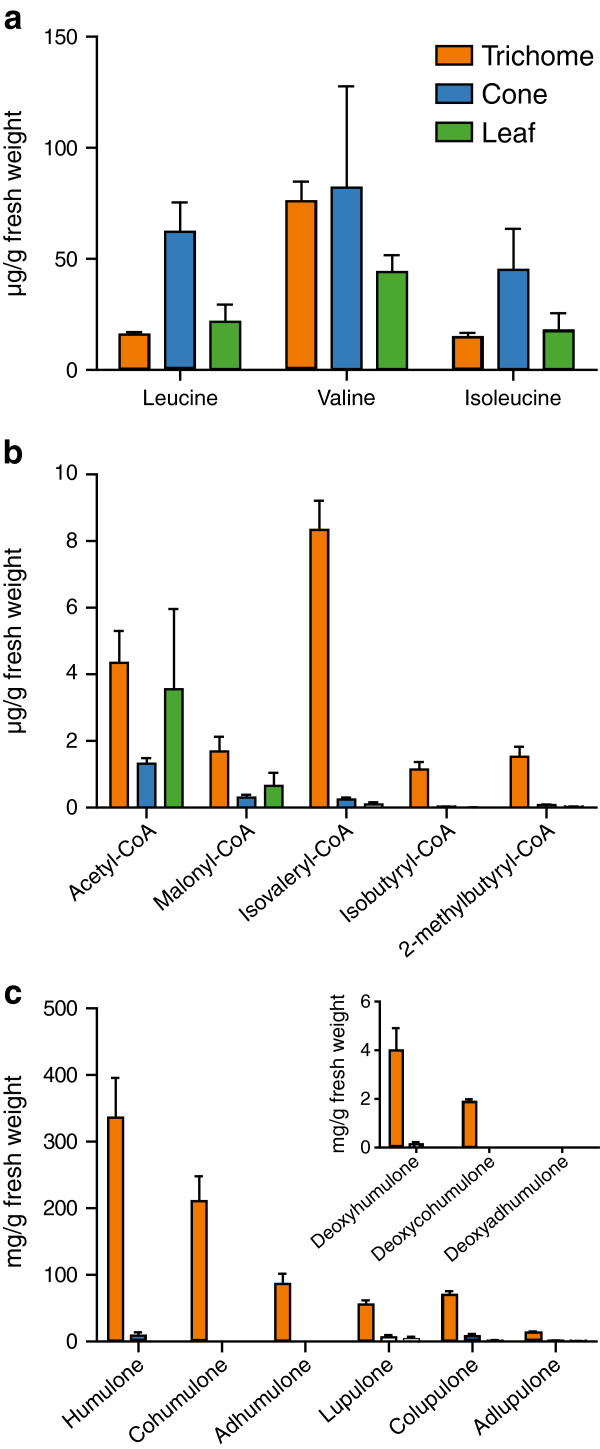
**Metabolite analysis of lupulin glands, leaves and cones.a**) Amounts of BCAAs (leucine, valine and isoleucine). **b**) Amounts of acyl-CoA precursors for bitter acid biosynthesis. **c**) Amounts of major bitter acid products. Inset shows amounts of diprenylated acylphloroglucinol intermediates. For all analyses, values represent mean ± standard error (n=5 for glands, n=3 for cone and leaf).

We quantified BCAA levels in lupulin glands, cones and leaves using HPLC with fluorescence detection of their derivatized adducts. Leucine, valine and isoleucine levels were similar in lupulin glands and leaves, with valine the most prominent (Figure 3a). BCAA levels in the cone tissue were highly variable and, in the case of leucine and isoleucine, were also elevated compared to glands and leaves.

Using a sensitive LC-MS method with multiple reaction monitoring, we measured the abundance of the acyl-CoAs that are used as substrates in bitter acid biosynthesis (malonyl-CoA, isovaleryl-CoA, isobutyryl-CoA and 2-methylbutyryl-CoA). Although acetyl-CoA is not used by VPS directly, it is converted to malonyl-CoA by acetyl-CoA carboxylase and therefore we included it in the analysis. Both acetyl-CoA and malonyl-CoA exhibit elevated levels in lupulin glands (Figure 3b). The starter substrates for the VPS reaction, isovaleryl-CoA, isobutyryl-CoA and 2-methylbutyryl-CoA, accumulated in glands while remaining largely absent in other tissues. Isovaleryl-CoA was five times more abundant than the other branched-chain acyl-CoAs.

HPLC analysis of the major bitter acids and their immediate precursors showed, as expected, that the α-acids accumulated to higher levels than the β-acids and that humulone was the most abundant end-product (Figure 3c). These results correlated well with the levels of acyl-CoA precursors (Figure 3b). We also quantified the diprenylated precursors (e.g. deoxyhumulone) of the bitter acids. Small amounts of deoxyhumulone and deoxycohumulone were present while deoxyadhumulone could not be detected (Figure 3c, inset). The absence of deoxyadhumulone may reflect the overall lower levels of isoleucine-derived bitter acids. Although we attempted to identify the products from the VPS reaction such as phlorisovalerophenone, as well as the monoprenylated forms, these metabolites were undetectable.

### HlBCAT1 and HlBCAT2 catalyze aminotransferase reactions with BCAAs

To further investigate BCAA metabolism in lupulin glands, we characterized the biochemical properties of the two gland-specific BCAT enzymes. HlBCAT1 is 393 amino acids long with calculated molecular weight of 43.2 kDa; HlBCAT2 is a 44.5 kDa (408 amino acid) protein. Nucleotide sequences for both enzymes are available in GenBank *HlBCAT1*, JQ063073; *HlBCAT2*, JQ063074]. The mature forms (i.e. lacking predicted transit peptides) of HlBCAT1 and HlBCAT2 were expressed as his-tagged recombinant proteins in *E. coli* and purified. Enzyme assays were performed in both the forward (anabolic, e.g. leucine to 2-ketoisocaproate) and reverse (catabolic, e.g. 2-ketoisocaproate to leucine) directions to test catalytic activities and to determine kinetic parameters (Table 2). Both enzymes functioned as BCATs and showed reversibility, as would be expected based on their ping pong reaction mechanism [27]. Unlike previous work in tomato, where the BCATs exhibited kinetic preferences corresponding to anabolic or catabolic functions [15], the hop BCATs had similar *K*_m_ values in either direction.

**Table 2:** Kinetic parameters for recombinant HlBCAT1 and HlBCAT2 enzymes in anabolic (forward) and catabolic (reverse) directions

**HlBCAT1**	**K**_**m**_**(μM)**^a^	**V**_**max**_**(μmol/mg/min)**^a^
**Anabolic:**		
Glutamate	720 ± 55.59	5.59 ± 0.13
Ketoisocaproate	35 ± 3.27	4.50 ± 0.04
Ketoisomethylvalerate	99 ± 10.75	2.95 ± 0.09
Ketoisovalerate	99 ± 6.53	4.23 ± 0.08
**Catabolic:**		
2-Oxoglutarate	30 ± 5.39	4.50 ± 0.16
Leucine	40 ± 9.20	4.84 ± 0.23
Isoleucine	230 ± 26.94	19.40 ± 0.85
Valine	340 ± 90.94	12.60 ± 0.96
**HlBCAT2**		
**Anabolic:**		
Glutamate	690 ± 88.51	135.90 ± 6.03
Ketoisocaproate	120 ± 11.43	119.20 ± 2.97
Ketoisomethylvalerate	160 ± 11.63	84.68 ± 1.70
Ketoisovalerate	200 ± 11.72	96.38 ± 1.45
**Catabolic:**		
2-Oxoglutarate	210 ± 58.86	36.92 ± 2.68
Leucine	280 ± 69.56	43.43 ± 2.55
Isoleucine	250 ± 41.07	88.08 ± 3.83
Valine	130 ± 27.74	23.29 ± 1.16

^a^mean ± standard error (n=3).

### Subcellular localization of hop BCATs

**Figure 4:**
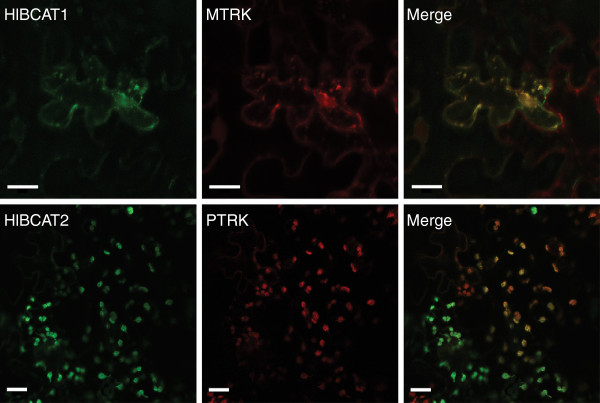
**Subcellular co-localization of BCAT signal peptides fused to GFP with organelle specific markers using transient expression in *Nicotiana benthamiana *leaves.** The HlBCAT1 signal peptide fused to GFP colocalized with the mitochondrial MTRK marker (upper panel) while the HlBCAT2 signal peptide-GFP fusion colocalized with the plastidial PTRK marker (lower panel). Plasmid constructs in Agrobacterium were agro-infiltrated into *N. benthamiana* leaves and fluorescent protein expression visualized using confocal microscopy. Scale bars represent 20 μm.

HlBCAT1, HlBCAT2 and HlBCAT3 contain putative N-terminal signal peptides while HlBCAT4 does not. TargetP [28] analysis predicted that HlBCAT1 and HlBCAT3 are localized to the mitochondria, and HlBCAT2 to the plastids. We tested the subcellular localization of the two BCATs with gland-specific expression, HlBCAT1 and HlBCAT2, using transient expression in *Nicotiana benthamiana* leaves. Hop leaves were not used for this experiment because they proved difficult to infiltrate with Agrobacterium solution. HlBCAT1-GFP constructs with signal peptide (Figure 4) or signal peptide with mature protein (Additional file 5: Figure S2) co-localized with the mitochondrial MTRK marker [29]. The full-length HlBCAT2 failed to localize to a recognizable compartment, however its signal peptide fused to GFP colocalized with the plastid PTRK marker (Figure 4, Additional file 5: Figure S2). HlBCAT1 and HlBCAT2 constructs without signal peptide accumulated in the cytosol (Additional file 5: Figure S2).

### Phylogenetic analysis of hop BCATs

**Figure 5:**
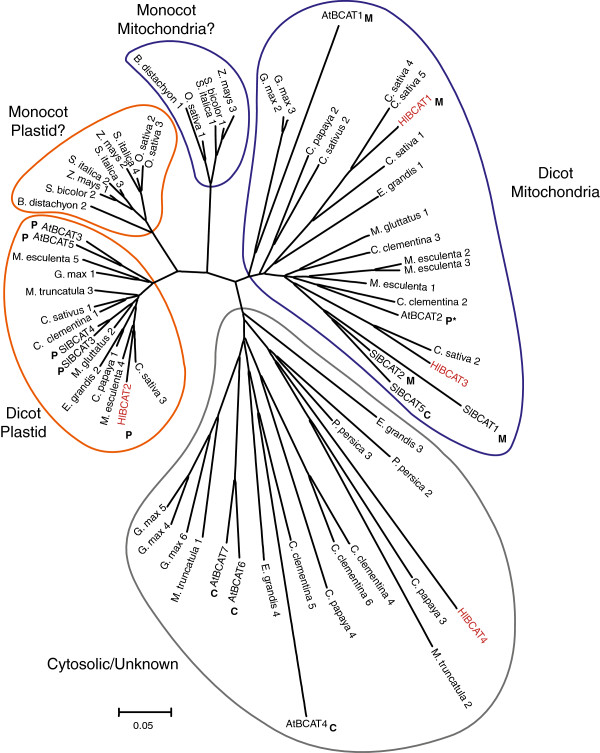
**A phylogenetic tree of BCAT proteins in plants constructed using the neighbour-joining method.** BCATs group into plastidial (orange line) and mitochondrial (blue line) clades, with apparent division into dicot and monocot subclades. A third clade (grey line) includes BCATs that fall into neither group. BCATs with experimentally-confirmed organelle localization are labelled: P, plastid; M, mitochondrion; C, cytosol. Species abbreviations are found in Methods (At, *Arabidopsis thaliana*; Hl, *Humulus lupulus*; Sl, *Solanum lycopersicon*). *As we note in the Discussion, the plastid localization of AtBCAT2 requires further analysis.

A phylogenetic tree was constructed using BCAT protein sequences from hop, Arabidopsis, tomato, *Cannabis sativa* and other plants obtained using the Phytozome database [22]. We observed a separation of dicot BCATs into two clades, which could be classified as biosynthetic/plastidial and catabolic/mitochondrial based on the presence of experimentally-localized proteins in each group (Figure 5). HlBCAT1 and 3 grouped into the mitochondrial BCAT clade and HlBCAT2 grouped into the plastidial clade, which agrees with their predicted localizations by TargetP and our analysis of intracellular localization (Figure 4). HlBCAT4 was grouped with the other BCATs which do not appear to localize to either the plastid or mitochondrion. Although the monocot BCATs clustered into two distinct groups that were positioned between the dicot clades, there is no experimental localization data for monocot BCATs at this time.

## Discussion

### Illumina sequencing provides new genomic resources for hop

Although the transcriptome of lupulin glands has been extensively studied using ESTs obtained through Sanger sequencing [5,8,30] and several 454 pyrosequencing datasets are available in NCBI, an in-depth comparison of the lupulin gland transcriptome with green tissues (i.e. leaves, cones) has not been reported. Schilmiller *et al*. [31] previously used 454 pyrosequencing to compare gene expression in tomato trichomes, leaves and stems, and RNA-seq analysis was used to identify terpene synthases in tomato trichomes [32]. Microarrays have also been used to compare the trichome and stem transcriptome of alfalfa [33]. The Illumina sequences reported here significantly expand the genomic resources available for hop, in particular leaves and cones for which limited sequences are available. The high number of contigs in the de novo transcriptome assembly probably relates to the presence of “broken contigs”, as evidenced by the relatively short contig length (745 bp). This may have resulted from our use of combined RNA-seq data from several hop cultivars, the lack of sufficient sequencing depth and the presence of introns from alternative splicing events in some transcripts. In addition to the further development of transcriptome resources for hop, an important next step is to link the transcriptome with a whole genome sequence and genetic map. However, the estimated 2.8 Gbp size of the *Humulus lupulus* genome [34] and the highly heterozygous nature of this dioecious species present obvious challenges for genome assembly.

### Lupulin glands synthesize precursor metabolites for bitter acid production

The expression pattern for genes related to photosynthesis (e.g. Calvin cycle), and the absence of bitter acids from the cone minus gland samples (Figure 3c), indicates that separation of the non-photosynthetic glands from photosynthetic cones was largely complete. Analysis of gene expression for primary metabolic pathways (TCA cycle and glycolysis) showed the reliance of lupulin glands on surrounding green tissues for the supply of sugars. The genes involved in the Calvin cycle were more abundant in the leaves and cones compared to lupulin glands (Figure 2a). The only exceptions to this pattern were genes encoding enzymes that function in both the Calvin cycle and glycolysis. The TCA cycle had similar expression levels across the leaf, cone and lupulin gland samples which can be attributed to its role in supplying intermediates for many pathways. Among the glycolysis transcripts three genes (*fructokinase*, *neutral/alkaline invertase* and *sucrose synthase*) stood out for their high expression in the lupulin glands compared to leaves. All three are involved in sucrose mobilization, underscoring the exogenous supply of sugars to the lupulin gland. This is in agreement with the previous suggestion that sucrose would be an ideal energy source and precursor for secondary metabolism in glandular trichomes [6].

The high amounts of bitter acids produced by elite hop cultivars represents a large carbon commitment within the lupulin glands. Given that sugars are imported, we analyzed the RNA-seq dataset to determine if other precursors (e.g. BCAAs and prenyl diphosphates) are imported or synthesized in situ. Both the MEP and BCAA pathways were more abundantly represented in the lupulin glands (Figure 2b), a finding that provides evidence for the de novo biosynthesis of BCAAs and DMAPP in these cells. BCAA biosynthesis in lupulin glands is logical given the demand for the acyl side-chains of the BCAAs but not the amino group, and the need to prevent the accumulation of excess nitrogen. This finding is in agreement with Schilmiller *et al.*[6] who noted that while phenylpropanoid-derived metabolites are synthesized in trichomes (e.g. phenylpropenes in basil) the import of phenylalanine into glandular trichomes was minimal.

Transcripts corresponding to the enzymes of the polyketide portion of the bitter acid pathway (e.g. *VPS*) were among the most abundant in the glandular trichome dataset (Figure 2b). The presence of two highly expressed aromatic prenyltransferases is notable since HlPT1 has only been shown to catalyze the first prenylation step in bitter acid biosynthesis [21]. The α- and β-acids require two and three prenyl groups, respectively, and the second prenyltransferase enzyme (HlPT2, 45% amino acid identity to HlPT1) may catalyze these steps.

### Lupulin gland-specific expression of transcription factors

RNA-seq analysis also revealed a number of lupulin gland-specific transcription factors which may be involved in regulating bitter acid biosynthesis or other metabolic or developmental processes in glandular trichomes (Table 2). Matouček and co-workers have identified several transcription factors that may control flavonoid biosynthesis in hop through the regulation of chalcone synthase (CHS) (e.g. HlMYB1, HlMYB2, HlMYB3, HlMYB7, HlbHLH2 and HlWDR1 [35-37]). Although all of these transcription factors could be identified in the hop transcriptome reported here, only transcripts of *HlMYB7* and *HlbHLH2* were more abundant in lupulin glands compared to leaves (Additional file 3: Table S3). This expression pattern makes sense since CHS, which plays a role in both the gland-specific production of xanthohumol and in the formation of flavonoids, is expressed throughout the plant. Indeed our RNA-seq analysis shows that *CHS_H1* is expressed in lupulin glands and leaves (Additional file 2: Table S2), with the three-fold higher expression levels in glands likely reflecting the production of xanthohumol. Two MYB transcription factors, CAN883 and CAN778, have been shown to be highly expressed in cannabis glandular trichomes and implicated in regulating trichome-specific metabolic processes [38]. Both have close homologs to hop transcription factors that are over ten-fold more abundant in the lupulin glands (Table 2). CAN738, which has 70% amino acid identity to the hop MYB protein encoded by comp63432_c0_seq1, was proposed to be involved in regulating cannabinoid formation. Since *Cannabis* and *Humulus* are closely related genera with glandular trichome-localized polyketide pathways, comp63432_c0_seq1 may be a candidate for regulating the bitter acid pathway.

### Lupulin glands contain both mitochondrial and plastidial BCATs

The biosynthesis and subsequent degradation of the BCAAs requires both metabolic and catabolic BCATs. The lupulin gland datasets contained two BCATs, *HlBCAT1* and *HlBCAT2*, which were abundant in the glandular trichomes. Based on RNA-seq and qRT-PCR analysis (Figure 2b, Additional file 4: Figure S1) *HlBCAT1* is exclusively expressed in the lupulin glands. Transient expression of the HlBCAT1-GFP fusion construct confirmed its mitochondrial targeting (Figure 4). HlBCAT2 with N-terminal signal peptide failed to localize to the plastid and appeared to form aggregates in the *N. benthamiana* epidermal cells (Additional file 5: Figure S2), however, the predicted HlBCAT2 signal peptide fused to GFP was targeted to the plastid (Figure 4). These localizations were further supported through the construction of a phylogenetic tree comparing BCATs from a variety of plant species (Figure 5). Problems with the experimental localization of hop plastid proteins have been previously reported for a GPP synthase small subunit (GPP-ssu) identified in lupulin glands [8].

### Kinetic analysis suggests lack of specialization for hop BCATs

Kinetic analysis has provided equivocal evidence for the catalytic specialization of BCATs for BCAA biosynthesis or catabolism. In tomato, biosynthetic BCATs (SlBCAT3 and 4) had K_m_ values with the keto acids that were an order-of-magnitude less than the catabolic enzymes (SlBCAT1 and 2), indicating specialization of the former for forward (biosynthetic) reactions [15]. In contrast, Arabidopsis AtBCAT1, which has been shown to be a catabolic enzyme, had K_m_ values for the keto acids (0.036-0.8 mM) that were much lower than for BCAA substrates (0.4-6 mM), suggesting a preference for the biosynthetic reaction. Indeed, the K_m_ values for AtBCAT1 with the keto acids were lower than those reported with the plastidial, biosynthetic enzyme AtBCAT3 [39]. The kinetic properties of the hop BCATs appear to be more similar to the Arabidopsis enzymes. The catabolic enzyme HlBCAT1 exhibited lower K_m_ values for keto acids, and therefore an apparent preference for the forward (biosynthetic) reaction, compared with than HlBCAT2 (Table 2). It is noteworthy that HlBCAT1 showed a lower K_m_ for leucine compared with valine and isoleucine which may reflect this enzyme’s preferred role in synthesizing isovaleryl-CoA for humulone biosynthesis. The lower V_max_ values for the HlBCAT1 may also explain why it is more highly expressed compared to HlBCAT2 in the lupulin gland tissue (Figure 2).

### Phylogenetic analysis indicates separation of biosynthetic and catabolic BCATs

To further analyze the function of the hop BCATs, we constructed a phylogenetic tree of diverse BCATs including well characterized enzymes from Arabidopsis and tomato. This analysis was successful in separating biosynthetic (plastidial) and catabolic (mitochondrial) BCATs into separate clades. BCATs are also reported to accumulate in alternative cellular compartments such as the cytosol and vacuole for an as of yet unknown function [15,16] and these genes likely make up the third group that includes several cytosolic enzymes and HlBCAT4 (Figure 5). An anomalous grouping was observed for AtBCAT2, which has been shown to be plastid localized [6] but which was placed in the mitochondrial clade. A previous study found *AtBCAT2* to be the only Arabidopsis gene to be co-expressed with other genes involved in BCAA catabolism [40], a role one would expect to be occupied by a mitochondrial BCAT. In addition, TargetP analysis of AtBCAT2 predicts mitochondrial rather than plastidial localization.

## Conclusions

This study provides an in-depth analysis of the transcriptional activity of primary and specialized metabolism leading to hop bitter acids. RNA-seq analysis showed that both the MEP and BCAA pathways were highly expressed in lupulin glands together with genes required for remobilization of carbon from sucrose. Branched-chain acyl-CoAs and bitter acids are present in higher levels in isolated lupulin glands compared with cones (with glands removed) and leaves. A plastidial BCAT enzyme involved in BCAA biosynthesis and a mitochondrial BCAT catalyzing BCAA degradation both show lupulin gland-specific expression. Together these findings indicate that the glandular trichomes are the site of biosynthesis for the precursors for bitter acid biosynthesis. The deep transcriptome sequencing reported here significantly expands the genomic resources available for hop. This new information, and our analysis of BCAA metabolism, will be useful for the further elucidation of enzymes and regulatory proteins involved in bitter acid production and for breeding hop cultivars with increased or modified bitter acid content.

## Methods

### Hop transcriptome assembly and RNA-seq analysis

RNA was isolated from lupulin glands sampled from hop cultivars ‘Taurus’, ‘Nugget’ and ‘Magnum’ (collected in Mainburg, Germany in August 2005), from ‘Taurus’ (collected in Saskatoon, SK in September 2011) and ‘Apollo’ (collected in Yakima, WA in August 2009). RNA was isolated from leaves and cones-minus-glands from ‘Apollo’ (collected Yakima 2009) and ‘Taurus’ (collected Mainburg 2005 and Saskatoon 2011). Glands were separated from cones by stirring in liquid nitrogen followed by filtration through a 1 mm screen to separate cone material. Total RNA was isolated using a CTAB method and an RNeasy kit with on-column DNaseI treatment (Qiagen) [41]. RNA was prepared for sequencing by the National Research Council Canada DNA Technologies Unit using the Illumina TrueSeq RNA sample preparation platform v.2 with multiplex labeling and subjected to paired-end sequencing using an Illumina HiSeq 2000 with a read length of 101 nt. Trimmomatic 0.15 [42] was used to remove adaptor sequences and trim bases with quality lower than 20 (Phred 33 quality scores). The transcriptome assembly was performed with Trinity [23] and refined using cd-hit-EST [24] to combine identical reads. CLC bio v.5 was used for RNA-seq analysis and statistical analysis via the Baggerly’s test [43]. Annotation of the transcriptome was performed using Blast2GO [44].

The Illumina data for all samples have been deposited in NCBI under Bioproject PRJNA175602 with the transcriptome available via accession number GAAW00000000. RPKM values for all transcripts is found in Additional file 6: Table S4.

### qRT-PCR analysis

cDNA was synthesized from 0.5 μg total RNA from lupulin glands of ‘Taurus’ and ‘Nugget’ sampled in Mainburg, Germany and ‘Taurus’ sampled in Saskatoon, Canada. RNA was isolated from leaves from ‘Taurus’ sampled in Mainburg, Germany in 2005, Saskatoon, Canada and ‘Apollo’ from Yakima, USA using the QuantiTect reverse transcription kit (Qiagen). Primers (Additional file 7: Table S5) were designed to amplify products of 101–110 bp. PCR reactions were performed with a StepOnePlus real-time PCR system (Applied Biosystems) using the QuantiFast SYBR Green qPCR kit (Qiagen). β-tubulin was used a reference gene. StepOnePlus software was used to calculate ΔΔC_T_ values [45].

### Metabolite analysis

For BCAA analysis, samples were weighed and ground in 5 vol (based on 1 mL per mg fresh weight of plant material) of 0.1 N HCl, centrifuged at 20,000 *g* for 10 min and derivatized using the AccQ-Tag reagent (Waters). Derivatized amino acids were analyzed with a Waters 2695 separation module using a Waters Symmetry C18 column (5 μm, 2.1 × 100 mm) and a Waters 2475 fluorescence detector as described by Cohen and Michaud [46]. Amino acids were quantified using standard curves.

For CoA analysis, samples were ground in liquid nitrogen and extracted twice with 10 vol of 5% TFA with internal standards (^13^C_3_ malonyl-CoA, butenoyl-CoA and benzoyl-CoA). Samples were evaporated, resuspended in 900 μL of acetonitrile: isopropyl alcohol: 100 mM KH_2_PO_4_: aqueous acetic acid (9:3:4:4, v/v/v/v) and solubilized by vortexing and sonication. Oasis WAX weak anion exchange cartridges (Waters) were primed with methanol, equilibrated with acetonitrile: isopropyl alcohol: water: acetic acid (9:3:4:4, v/v/v/v), the samples loaded and washed with the equilibration mixture. Samples were eluted with 250 mM ammonium hydroxide in 80% methanol, dried and stored at −20°C. The residue was reconstituted in 100 μL of water: acetonitrile (95:5, v/v) containing 5 mM triethylamine (TEA) and 3 mM acetic acid, vortexed, sonicated and centrifuged. The injection volume was 10 μL. CoAs were separated on a Waters Acquity UPLC system with a Waters BEH C18 column (1.8 μm, 2.1 × 100 mm) with a Vanguard guard column. Mass detection used a Quattro Ultima (Waters) in positive ion, multiple reaction monitoring (MRM) mode. Solvents were A=5 mM TEA and 3 mM acetic acid in water, and B=5 mM TEA and 0.3 mM acetic acid in acetonitrile: water (95:5, v/v). A flow rate of 200 μL/min was used with an initial solvent composition of 98:2 A:B changing to 87:13 A:B over 19 min. Detection of the acyl-CoAs using MRM involved neutral loss of 507 Da from each protonated molecular ion [47]. Quantification by standard curves used MassLynx 4.1 (Waters). Since there was no standard for 2-methybutyryl-CoA, quantification was based on the calibration curve constructed for n-valeryl-CoA.

For polyketide analysis, samples were ground in 10 vol of 80% MeOH, filtered through a 0.45 μm Spin-X filter (Corning) and separated on a Waters 2695 separation module using a Waters Symmetry C18 column (5 μm, 2.1 × 100 mm). A flow rate of 0.3 mL/min was used with an initial solvent composition of acetonitrile: water (46:54, v/v) with 0.05% formic acid changing to acetonitrile: water (95.5:4.5, v/v) with 0.05% formic acid over 25 min. Metabolites were detected using a Waters photodiode array detector at 237 nm and 3100 Mass Detector in SIR with positive electrospray and a cone voltage of 30 V. Metabolite levels were determined with standard curves of deoxyhumulone, humulone and lupulone.

### BCAT characterization

Oligonucleotides used in cloning procedures are found in Additional file 7: Table S5. Signal peptides were predicted using Mitoprot [48] and ChloroP [49]. The open-reading frames of HlBCAT1 and HlBCAT2 lacking signal peptides were amplified using *Pfu* Ultra II polymerase (Agilent), the amplification products cloned into pCR8/GW/TOPO (Invitrogen) and recombined into pHIS8/GW using LR Clonase (Invitrogen). HlBCAT1 and HlBCAT2 in pHIS8/GW were transformed into *E. coli* BL21(DE3) Rosetta2 pLysS cells (Novagen). Cells were grown in Luria-Bertani media containing 50 μg/L kanamycin and 34 μg/L chloramphenicol to an OD_600_ of 0.6 at 37°C, cooled to 18°C, induced with 0.3 mM IPTG and incubated for 4 h at 18°C. Bacterial cultures were pelleted and frozen at −80°C. Pellets were thawed, resuspended in 20 mM Tris HCl (pH 8.0), 300 mM NaCl, 100 μM pyridoxal-5-phosphate (PLP), 10% glycerol, 5 mM imidazole, 5 mM β-mercaptoethanol, 0.2% triton X-100, 0.1% CHAPS, 0.2% sarkosyl, 0.5 mM PMSF, 1 μg/ml pepstatin A and 1 μg/ml leupeptin, and lysed by sonication. Recombinant proteins were purified from the cell lysate using Talon resin (Clontech). The resin-bound protein was washed with 20 mM Tris HCl (pH 8.0), 300 mM NaCl, 10% glycerol, 10 mM imidazole, 5 mM β-mercaptoethanol and eluted with the same buffer composition containing 150 mM imidazole. Samples were desalted and concentrated using Amicon Ultra filters (Millipore) and stored at −80°C. Protein concentration was determined using the Bradford assay [50] and protein purity was assessed at >95% via SDS-PAGE gel analysis with Coomassie brilliant blue stain.

Anabolic reactions were linked to glutamate dehydrogenase to allow monitoring with NADH turnover as described by Prohl *et al*. [51]. Anabolic assays contained 66 μg/ml HlBCAT1 or 12 μg/ml HlBCAT2, 250 mM Tris HCl (pH 8.5), 50 μM PLP, 1.5 mM dithiothreitol, 0.2 mM EDTA, 100 mM ammonium chloride, 0.2 mM NADH, 3 units/mL glutamate dehydrogenase (Sigma), 2 mM glutamate for branched-chain keto-acid kinetics and 0.5 mM ketoisovalerate for glutamate kinetics. Assays were run in triplicate at 22°C and observed in real time at 340 nm with an Ultraspec 3100 pro spectrophotometer (Biochrom). Time point assays were employed for catabolic reactions. Catabolic assays contained 5.4 μg HlBCAT1 or 1.2 μg HlBCAT2, 20 mM Tris HCl (pH 8.5), 50 μM PLP, 1.5 mM dithiothreitol, 0.2 mM EDTA, 2 mM 2-oxoglutarate for BCAA kinetics and 2 mM leucine for 2-oxoglutarate kinetics. Assays were run in triplicate at 22°C for 2 min and halted with the addition of HCl. Samples were centrifuged at 20,000 *g* for 10 min, derivatized using the AccQ-Tag reagent (Waters) and analyzed as described above.

### Analysis of subcellular localization

Three constructs in pCR8/GW/TOPO were prepared for each gene: full-length including predicted signal peptide, the mature protein lacking the signal peptide, and the signal peptide only. Each was recombined into pEarleyGate103 to generate C-terminal GFP fusion constructs under the control of the 35S promoter [52]. Plasmid constructs of HlBCATs, and mitochondrial (MTRK) or plastid (PTRK) markers [29], were transformed into Agrobacterium C58 and infiltrated into *Nicotiana benthamiana* leaves as previously described [53]. Plants were grown for 2–4 days and the leaves directly observed with a Carl Zeiss Axiovert 200M/ConfoCor 2/LSM 510 confocal laser scanning microscope. GFP was observed with an argon laser with excitation at 488 nm and emission at 505–530 nm. mCherry was observed with a helium laser with excitation at 543 nm and emission at 560–615 nm.

### Phylogenetic tree

BCAT protein sequences for were obtained from the Phytozome database [22] for *Manihot esculenta*, *Populus trichocarpa*, *Cucumis sativus*, *Prunus persica*, *Medicago truncatula*, *Glycine max*, *Carica papaya*, *Citrus clementina*, *Eucalyptus grandis*, *Mimulus guttatus*, *Sorghum bicolor*, *Zea mays*, *Setaria italica*, *Oryza sativa*, and *Brachypodium distachyon*. Previously characterized Arabidopsis and tomato proteins were included along with those from the *Cannabis sativa* genome [54]. All sequences were aligned using ClustalW [55] with variable N-terminal regions removed when present. Mega5 [56] was used to construct a neighbour-joining tree with 10,000 bootstrap replicates.

## Abbreviations

BCAA: Branched-chain amino acid; BCAT: Branched-chain aminotransferase; BCKDH: Branched-chain ketoacid dehydrogenase complex; CHAPS: 3-[(3-cholamidopropyl)dimethylammonio]-1-propanesulfonate; CoA: Coenzyme A; DMAPP: Dimethylallyl diphosphate; EDTA: Ethylenediaminetetraacetic acid; GFP: Green fluorescent protein; IPTG: Isopropyl β-D-1-thiogalactopyranoside; MEP: Methyl-D-erythritol 4-phosphate; qRT-PCR: Quantitative reverse transcription polymerase chain reaction; PLP: Pyridoxal-5-phosphate; PMSF: Phenylmethylsulfonyl fluoride; RPKM: Reads per kb exon model per million mapped reads; sp: Signal peptide; VPS: Valerophenone synthase.

## Competing interests

Partial funding for this work was provided by Hopsteiner Inc, a major hop processing company.

## Authors’ contributions

SMC isolated RNA for RNA-seq, assembled the hop transcriptome, analyzed transcriptome data, performed qRT-PCR analysis, characterized the BCATs, measured BCAA and bitter acid levels and wrote the manuscript. VV cloned and expressed BCATs. SA and RP quantified acyl-CoAs. JEP conceived of the study, and participated in its design and coordination and wrote the manuscript. All authors read and approved the final manuscript.

## Supplementary Material

Additional file 1 Table S1 Total Illumina sequencing reads for each RNA sample and the number of reads that mapped to the reference transcriptome. Click here for file

Additional file 2 Table S2 RNA-seq analysis of metabolic genes compared between lupulin gland and leaf, lupulin gland and cone (minus glands), and leaf and cone (minus glands). Data is represented in reads per kb exon model per million mapped reads (RPKM). Note that each tissue comparison is located on a separate Excel sheet. Click here for file

Additional file 3 Table S3 Transcript abundance of transcription factors compared between lupulin gland and leaf, lupulin gland and cone (minus glands) and leaf and cone (minus glands). Data is represented in RPKM. Click here for file

Additional file 4 Figure S1 Relative abundance of select metabolic transcripts in lupulin gland and leaf samples as determined by qRT-PCR. Data is presented using relative quantitation (RQ) based on ΔΔC_T_ values. Samples are normalized using β-tubulin. Data represents the mean and standard error, n=3. Transcript abundance between panels a, b and c is not directly comparable in this figure. **a**) qRT-PCR analysis of *HlBCAT1* and *HlBCAT2*. A single gland sample was used as reference. **b**) qRT-PCR analysis of genes encoding enzymes of the BCKDH complex (ketoacid dehydrogenase E1α and E1β, dihydrolipoyl acyltransferase (E2), and dihydrolipoyl dehydrogenase (E3)). A single leaf sample was used as reference. **c**) qRT-PCR analysis of *VPS* and *HlPT1*. A single leaf sample was used as reference. Click here for file

Additional file 5 Figure S2 Subcellular co-localization of GFP fusion proteins of HlBCAT1 and HlBCAT2, with and without N-terminal signal peptides, compared with organelle specific marker proteins using transient expression in *Nicotiana benthamiana* leaves. MTRK and PTRK are mitochondrial and plastidial makers. Scale bars represent 20 μm. Click here for file

Additional file 6 Table S4 RPKM values for lupulin glands, cones (minus glands) and leaves. Click here for file

Additional file 7 Table S5 Sequences of oligonucleotides used. Click here for file
