# Supplementary material for: Transcriptome analysis of bitter acid biosynthesis and precursor pathways in hop (Humulus lupulus)
Source: BMC Plant Biol. 2013 Jan 24;13:12. doi: 10.1186/1471-2229-13-12 (PMC3564914; doi:10.1186/1471-2229-13-12)
Supplement: Additional file 1 Table S1 — Total Illumina sequencing reads for each RNA sample and the number of reads that mapped to the reference transcriptome. [file 1471-2229-13-12-S1.docx]

| **Table S1 – Total sequencing reads for each RNA sample sequenced and the breakdown of reads that mapped to the reference transcriptome.**   \| **Tissue** \| **Mapped reads** \| **Unmapped**  **reads** \| **Total**  **reads** \| \| --- \| --- \| --- \| --- \| \| **Lupulin gland** \|  \|  \|  \| \| Taurus Germany \| 16,105,302 \| 1,502,826 \| 17,608,128 \| \| Taurus Saskatoon \| 13,644,745 \| 2,111,383 \| 15,756,128 \| \| Apollo \| 17,774,686 \| 1,496,368 \| 19,271,054 \| \| Nugget \| 22,371,660 \| 2,417,100 \| 24,788,760 \| \| Magnum \| 17,762,131 \| 1,658,739 \| 19,420,870 \| \| **Cone** \|  \|  \|  \| \| Taurus Germany \| 14,408,203 \| 1,301,361 \| 15,709,564 \| \| Taurus Saskatoon \| 17,900,191 \| 1,696,691 \| 19,596,882 \| \| Apollo \| 17,687,299 \| 1,376,365 \| 19,063,664 \| \| **Leaf** \|  \|  \|  \| \| Taurus Germany \| 23,413,984 \| 1,349,638 \| 24,763,622 \| \| Taurus Saskatoon \| 18,922,366 \| 1,299,480 \| 20,221,846 \| \| Apollo \| 16,210,251 \| 1,138,503 \| 17,348,754 \| |  |  |
| --- | --- | --- | --- | --- | --- | --- | --- | --- | --- | --- | --- | --- | --- | --- | --- | --- | --- | --- | --- | --- | --- | --- | --- | --- | --- | --- | --- | --- | --- | --- | --- | --- | --- | --- | --- | --- | --- | --- | --- | --- | --- | --- | --- | --- | --- | --- | --- | --- | --- | --- | --- | --- | --- | --- | --- | --- | --- | --- | --- | --- | --- | --- |
