# Supplementary material for: Transcriptome analysis of bitter acid biosynthesis and precursor pathways in hop (Humulus lupulus)
Source: BMC Plant Biol. 2013 Jan 24;13:12. doi: 10.1186/1471-2229-13-12 (PMC3564914; doi:10.1186/1471-2229-13-12)
Supplement: Additional file 4 Figure S1 — Relative abundance of select metabolic transcripts in lupulin gland and leaf samples as determined by qRT-PCR. Data is presented using relative quantitation (RQ) based on ΔΔCT values. Samples are normalized using β-tubulin. Data represents the mean and standard error, n=3. Transcript abundance between panels a, b and c is not directly comparable in this figure. a) qRT-PCR analysis of HlBCAT1 and HlBCAT2. A single gland sample was used as reference. b) qRT-PCR analysis of genes encoding enzymes of the BCKDH complex (ketoacid dehydrogenase E1α and E1β, dihydrolipoyl acyltransferase (E2), and dihydrolipoyl dehydrogenase (E3)). A single leaf sample was used as reference. c) qRT-PCR analysis of VPS and HlPT1. A single leaf sample was used as reference. [file 1471-2229-13-12-S4.pdf]

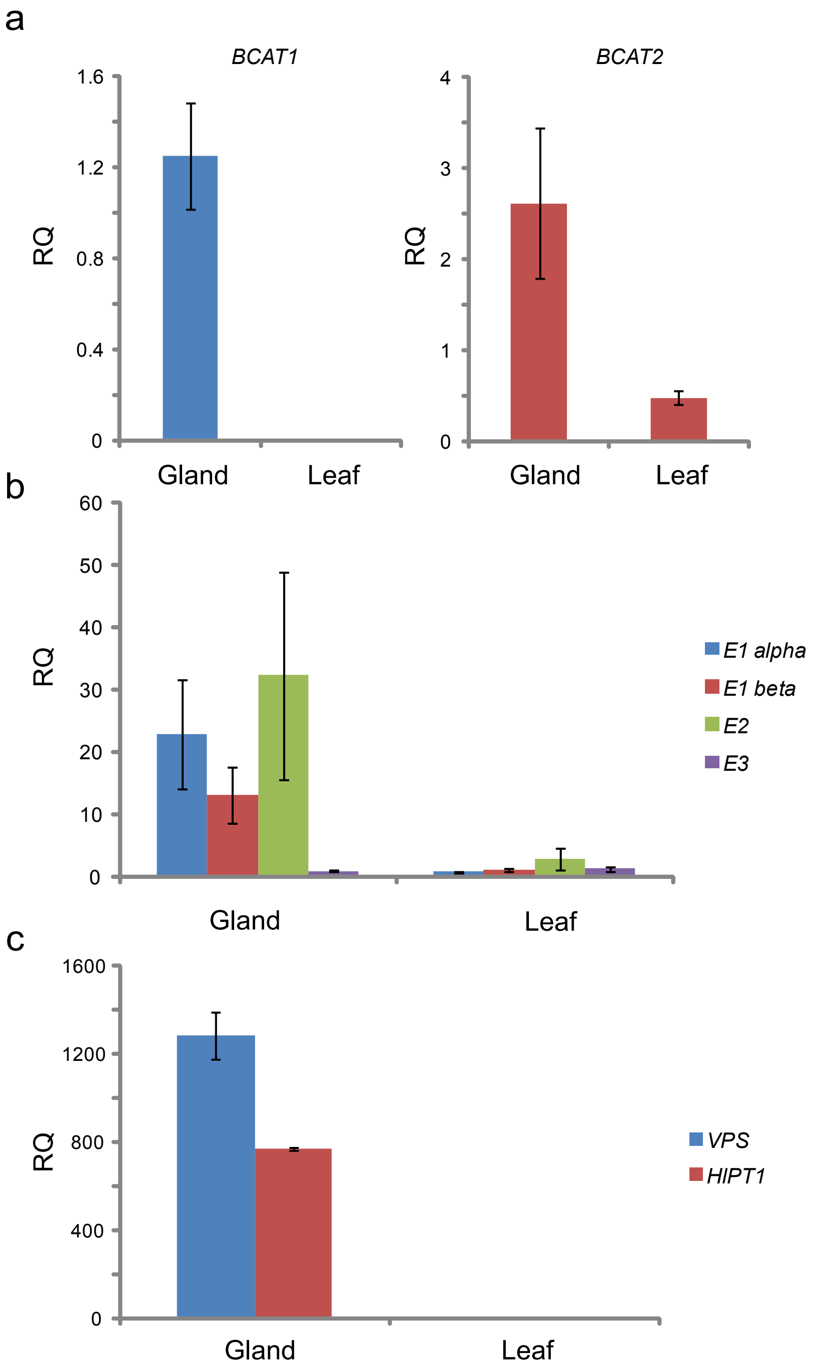

Figure S1 – Relative abundance of select metabolic transcripts in lupulin gland and leaf samples as determined by qRT-PCR. Data is presented using relative quantitation (RQ) based on $\Delta\Delta C_T$ values. Samples are normalized using β-tubulin.
a) qRT-PCR analysis of *HlBCAT1* and *HlBCAT2*. A single gland sample was used as reference.
b) qRT-PCR analysis of genes encoding enzymes of the BCKDH complex (ketoacid dehydrogenase E1α and E1β, dihydrolipoyl acyltransferase (E2), and dihydrolipoyl dehydrogenase (E3)). A single leaf sample was used as reference.
c) qRT-PCR analysis of *VPS* and *HlPT1*. A single leaf sample was used as reference.
Data represents the mean and standard error, n=3. Transcript abundance between panels a, b and c is not directly comparable in this figure.
