# Supplementary material for: Transcriptome analysis of bitter acid biosynthesis and precursor pathways in hop (Humulus lupulus)
Source: BMC Plant Biol. 2013 Jan 24;13:12. doi: 10.1186/1471-2229-13-12 (PMC3564914; doi:10.1186/1471-2229-13-12)
Supplement: Additional file 5 Figure S2 — Subcellular co-localization of GFP fusion proteins of HlBCAT1 and HlBCAT2, with and without N-terminal signal peptides, compared with organelle specific marker proteins using transient expression in Nicotiana benthamiana leaves. MTRK and PTRK are mitochondrial and plastidial makers. Scale bars represent 20 μm. [file 1471-2229-13-12-S5.pdf]

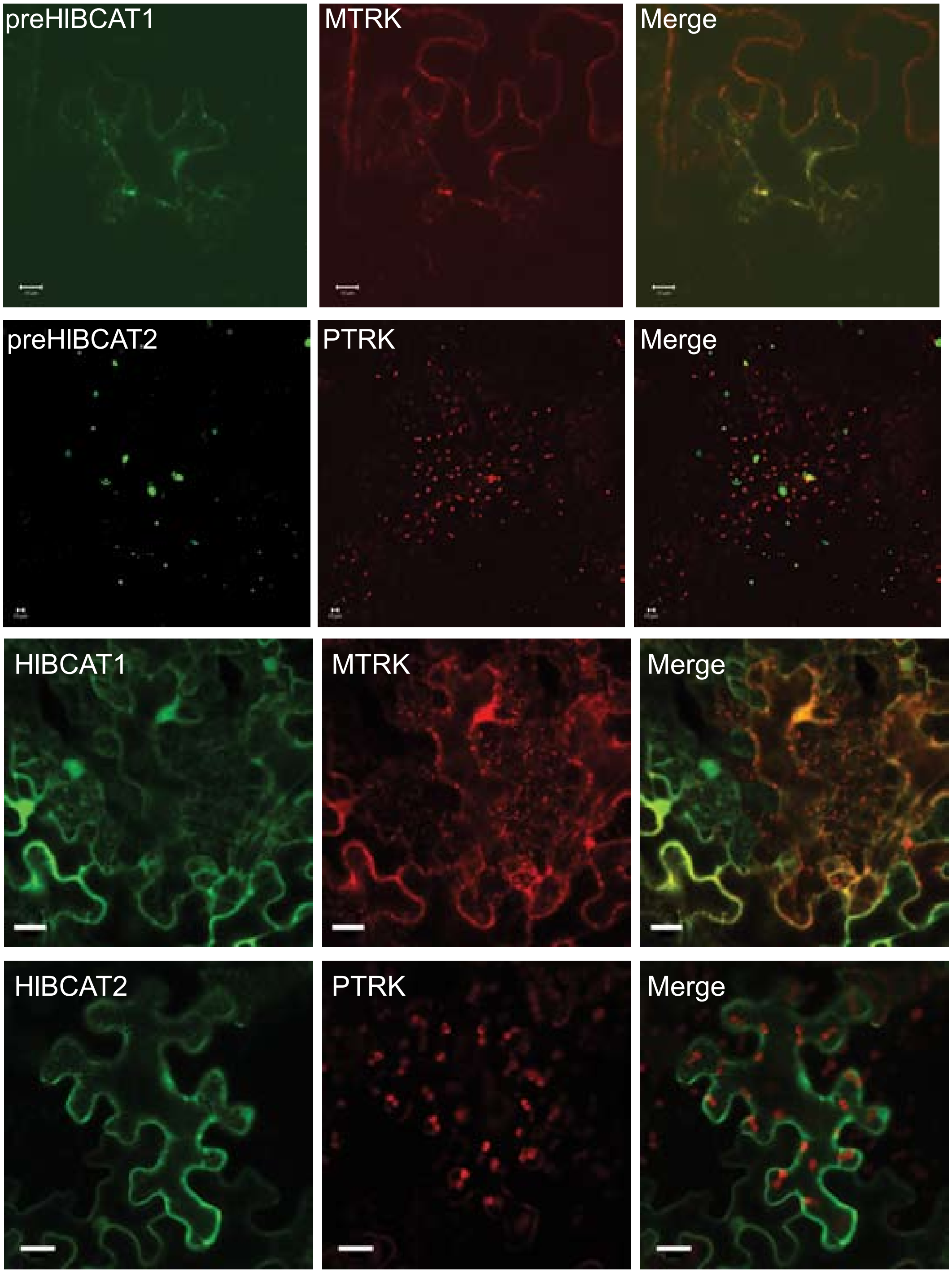

Figure S2 – Subcellular co-localization of GFP fusion proteins of HlBCAT1 and HlBCAT2, with and without N-terminal signal peptides, compared with organelle specific marker protesins using transient expression in *Nicotiana benthamiana* leaves. MTRK and PTRK are mitochondrial and plastidial makers. Scale bars represent 20 μm.
